# ‘What would Bandit do?’: reaffirming the educational role of Australian children’s television during the COVID-19 pandemic and beyond

**DOI:** 10.1177/1329878X20948272

**Published:** 2021-02

**Authors:** Jessica Balanzategui, Liam Burke, Joanna McIntyre

**Affiliations:** Swinburne University of Technology, Australia

**Keywords:** Australian children’s television, Australian content quotas, COVID-19, education, home-school education

## Abstract

The COVID-19 pandemic has highlighted the multifaceted socio-cultural functions of Australian children’s television. As social distancing measures forced school students to study from home, local children’s TV producers and distributors contributed to home-based learning. Yet, in response to the pandemic, the Federal Government has indefinitely suspended Australian children’s television quotas, the regulatory framework that sets minimum hours of local children’s content for commercial television broadcasters. In response to government imposed budgetary restraints, public broadcaster, the ABC, has also made redundances in its children’s content department. Such changes have occurred at a critical juncture in which the sector’s long-standing contributions to the education of Australian children and pedagogy of local teachers, caregivers and parents have been brought to the fore. We argue that this pedagogical function is a core but often overlooked element of the socio-cultural value of the sector that has been highlighted during the pandemic.

The COVID-19 pandemic has highlighted the multifaceted socio-cultural functions of Australian children’s television. As social distancing measures forced school students to study from home, local children’s TV producers and distributors contributed to home-based learning. Notably, in partnership with state and territory education departments, national broadcaster, the Australian Broadcasting Corporation (ABC), delivered curriculum-linked content and online resources to support home-schooling for caregivers, parents and teachers through its ABC Education portal, including teacher-led ‘mini lessons’. Moreover, ABC children’s shows such as *Bluey* and *Behind the News* (*BtN*) produced materials and content to keep Australian children informed during the pandemic, with *BtN* even providing Australian children the opportunity to have direct access to the Prime Minister to ask questions about the pandemic and Government’s response.

Nevertheless, children’s content was a key target of a recent round of redundancies at the ABC due to budgetary restraints: in June 2020, the ABC announced the closure of Melbourne-based children’s division ME TV and discontinuation of the programme *Definitely Not News* (*DNN*), with 15 staff in children’s content being made redundant (Fitzsimmons, 2020). Furthermore, in response to the pandemic the Federal Government has indefinitely suspended Australian children’s television quotas, the regulatory framework that sets minimum hours of local children’s content for commercial television broadcasters. Although these quotas have long supported a robust domestic children’s television sector, they have been under threat for some time, as commercial broadcasters have for many years advocated for their removal. Paul Fletcher, the Minister for Communications, Cybersecurity and the Arts, described the suspension of these quotas in April 2020 as ‘an emergency red tape reduction measure’ to support the financial interests of commercial broadcasters during the pandemic (cited in Quinn, 2020). Considering these disruptions to the production and distribution of Australian children’s television content, this essay examines the relationship and significance of Australian children’s television to national education. Responding to what could potentially become a crisis of a different sort, we argue that contributions to pedagogy and education are a core but often overlooked element of the socio-cultural value of the sector, but one that the pandemic has brought to light.

The public broadcasters, ABC and SBS, are not required to abide by Australian content quotas, instead producing local and children’s content under the requirements of their charters. The ABC, in particular, has been a key driver of quality local children’s content through its dedicated children’s TV channel, ABC ME, launched in 2009. However, budgetary restrictions have made the ABC’s track record of quality local children’s content increasingly difficult to sustain. Government analysis published in 2017 in response to a federal inquiry into local and children’s screen content raised concerns that the ABC may have ‘recently reduced its commitment to producing children’s content’ (Parliament of Australia, 2017: 57). The report raised the prospect of imposing quotas on the ABC and SBS to ‘ensure that quality programming will continue to be available for Australian children’ (Parliament of Australia, 2017: 57). Yet, fulfilling such obligations may be increasingly difficult due to Federal Government imposed funding cuts: notably, despite the ABC’s contribution to children’s education during lockdowns and home-schooling periods, the Federal Government has upheld a pause in indexation of ABC funding up until July 2022. Analysis has demonstrated that by end of the financial year (2020/2021), the ABC’s operational funding base will have been reduced by 10% since 2013 (Wijekumar, 2020). As Anna Potter and Huw Walmsley-Evans (2017) point out, the ABC’s local content targets were reduced to 25% (from 50%) in 2015, and the ABC is free to ‘pull funding from the children’s television budget whenever it wishes’, as has been highlighted by the recent children’s content redundancies and programme cancellations.

Taking these factors into account, our analysis in this essay builds upon recent work by Cunningham et al. (2016) that maps the contemporary shape of the market for educational screen content in Australia, in which they identify a ‘growing demand’ for and ‘increased use of screen content in formal education’ (p. 1). Rather than focusing on how the fluid and multi-layered screen education market operates, we articulate how educating Australian children in culturally specific ways has been a key agenda of the local children’s television sector for many decades – one that finds renewed relevance in the time of COVID-19. We begin by outlining how the foundations of this architecture were established, taking the much-loved Australian children’s show *Round the Twist* as a case study to highlight how deep and productive engagement between Australian children’s television, pedagogy and curriculum developed. We then investigate contemporary intersections between Australian children’s television and education by exploring how ABC shows *Bluey* and *BtN* contribute to the education of local children. We contend that both shows were well-placed to support home education during the initial nationwide ‘lockdown’ of the pandemic. In doing so, this essay highlights how even – or perhaps *especially* – during the pandemic, the Australian children’s television sector continues to contribute to the cultural life and education of Australian children. Yet, the complex infrastructure that underpins this socio-cultural function is currently in flux, which may challenge the sector’s future capacity in this regard.

## *Round the Twist*, Australian children’s television and curricula

In Australia, the production and distribution of local children’s television has long been underwritten by what Potter (2015) describes as ‘a set of public value principles’ (p. ix). These principles are formalised in a multifaceted policy settlement that includes the following:

1. The Australian Children’s Television Standards introduced in 1984.
2. Children’s content quotas on commercial free-to-air television, which ensure local children’s content is provided in key timeslots when children are likely to be watching. These programmes must be ‘made specifically for children [. . .] be entertaining and well produced with high production standards, and enhance a child’s understanding and experiences’ (Screen Australia, 2018).
3. The work of the Australian Children’s Television Foundation (ACTF, 2019) since their inception in 1982 to support a local children’s television sector that, in line with their mission statement, provides Australian children with ‘entertainment media made especially for them, which makes an enduring contribution to their cultural and educational experience’ (p. 46).
4. The ABC’s responsibility, outlined in its Charter, to broadcast ‘programs of an educational nature’ (ABC Act, 1983).

This unique policy settlement has meant that Australian children’s television has, since the 1980s, been closely aligned with the objectives of national, state- and territory-based curriculum, which is partly why the sector was well-placed to support home-based learning during the COVID-19 pandemic. As a key and formative example of the success of this settlement, the live-action fantasy drama programme *Round the Twist* highlights the productive relationships between the ACTF, television broadcasters, teachers and schools that undergird the educational contributions of Australian children’s television content. This programme – written by award-winning children’s author and educator Paul Jennings – was a flagship product of non-profit children’s content production and policy hub the ACTF in its first decade of operations. Notably, the ABC’s educational and curriculum-aligned activities in the domain of children’s content extend strategies pioneered by the ACTF, which has long developed curriculum-aligned teaching resources to accompany local children’s television shows. *Round the Twist* was broadcast on the ABC from Season 2, however, the first season of the programme aired on commercial broadcaster Seven Network, diminishing the commercial broadcasters’ current claims that the quotas are ‘completely irrelevant’ (Free TV Australia, Submission: 18). Commercial broadcasters, too, have played an important role in delivering local children’s content aligned with curriculum and educational aims.

*Round the Twist* is regarded by current ACTF CEO Jenny Buckland as the show that ‘set a standard’ for Australian’s children’s TV and remains a high water mark (cited in Guillaume, 2017), and it evidences deep engagements between the Australian curriculum and children’s television since the early 1990s. Numerous resources for teachers of Middle Primary and Middle Years students (ages 4–9) were produced to support lessons associated with screenings of the programme in class (with 40 resources for this age group currently collated on the ACTF website). These lessons and activities focus on a diverse array of study areas and learning outcomes aligned with curriculum, including Screen Literacy, Humanities and Social Sciences, Ethical Understanding and English. The show has thus become a template for ‘quality’ Australian children’s television that aims to fulfil a social function by making ‘an enduring contribution’ to the ‘cultural and educational experience’ of Australian children, in line with the ACTF’s mission statement.

As a result, *Round the Twist* became firmly embedded in the Australian curriculum for students aged 4–9 throughout the 1990s. This success is evident in contributions from teachers in Australian pedagogy journal *Screen Education*, in which they reflect on their *Round the Twist*-based lessons (Burton, 2005; Burton, 2007; Pearson, 2007). As Year 5 teacher Linda Pearson (2007) states, her teaching and learning activities planned around the series ensured that students ‘developed a deep knowledge and understanding of media, further developing their skills and ability for communication and constructing texts using media language’ (p. 82). Pearson (2007) concludes that her *Round the Twist*-based lessons were deemed a ‘“huge success” by the teachers, students and parents involved’ (p. 88). James Curzon, coordinator of a secondary school Media Studies programme, highlights how valuable the show and associated resources were for ‘teaching production and media techniques’ (cited in Burton, 2005: 98).

The influence of *Round the Twist* within and beyond the curriculum for a generation of Australian children is evidenced by the surge of publications and events expressing nostalgia for the programme throughout the 2010s. For instance, popular podcasts have been produced that reflect on the show’s legacy ( ‘The Original Bronson’, ABC 2016; ‘Tales from The Twists’, 2019), and news media organisations have published extensive oral histories (Guillaume, 2017). *Round the Twist* has come to exemplify productive intersections between local children’s television and the educational and cultural life of Australian children, and its curriculum-aligned teaching resources established an effective model for the ACTF, other local children’s television producers and broadcasters, and teachers. These are intersections that we argue have been realised in targeted, reinvigorated ways in response to the COVID-19 pandemic.

## *Behind the News* and current affairs for Australian children

The ABC’s *BtN* highlights how these long-standing alignments between Australian children’s television and pedagogy enabled an efficient pivot to supporting children’s learning during the pandemic. *BtN* first aired in 1968 and ran until 2003 before being reinstated in 2005.^1^ *BtN* currently airs on ABC ME, and has a considerable online presence that supports and highlights its intended pedagogical functions. The programme’s accompanying website includes a page devoted to teaching resources associated with each episode (ABC Behind the News, 2019). The website provides each of the show’s full ‘Classroom Episodes’ (designed to function as classroom or in-home lessons), as well as individual news story segments, downloadable study materials and additional activities for students. Activities include participatory opportunities such as ‘Ask a Reporter’: a weekly, live-stream Q and A session in which child viewers can ask *BtN* reporters questions in real time. The show’s remit is to provide children aged 8–13 years (upper primary and lower secondary school students) with age-appropriate explanations and information regarding contemporary news and current affairs, including issues specific to Australian children that may be overlooked in adult or global current affairs programmes. A number of studies – particularly in the fields of journalism and education – indicate that children’s engagement with news through social and mass media can increase not just their knowledge and awareness of politics, but also citizenship in a democracy (Gunter and Gunter, 2020; Nygren et al., 2019). *BtN*’*s* youth-focussed music, graphics, topics and tone are designed to appeal to its target audience in ways that intend to spark their interest in politics, society and world events.

At its peak, *BtN* ‘had well over a million viewers a week’ and ‘about 90 per cent of schools made use of the programme’ (Higgens, 2003). While it has been 52 years since the show first aired, the ABC’s (2019) Annual Report states that *BtN* continues to have ‘high engagement on broadcast and online platforms’ (p. 18), and cites it as one of its key offerings that contribute ‘practical digital literacy content for primary school-aged audiences’ (p. 131). Pavolic (2018) highlights quotes from teachers describing the program as an ‘excellent research starter’ that would be ‘hard to replace’ in Australian classrooms. *BtN* thus performs a number of functions as a classroom and in-home learning resource. In addition to being freely available on ABC ME and online, it is a screen product that forestalls any concerns teachers and parents may have about children being exposed to news content that may not be appropriate for younger audiences. To a similar end, *BtN* communicates directly to Australian children in ways that provide a culturally specific, contextualised perspective on global current affairs.

At-home learning contexts during the COVID-19 lockdown have brought the socio-cultural value of *BtN* to the fore. While news and current affairs were critical to public health and safety during this period, some of this news content would likely be distressing for children. In this milieu, *BtN* mitigated a bombardment of news stories about the pandemic by providing stories on a variety of alternative topics, while maintaining age-appropriate coverage of COVID-19-related issues. For example, one episode discussed what it means to ‘flatten the curve’ and how police in different countries were enforcing COVID-19 restrictions. Another featured Australian Prime Minister, Scott Morrison, at Parliament House answering *BtN* viewers’ video questions about COVID-19 and its social impacts. These examples demonstrate *BtN*’*s* capacity to give children access to locally contextualised information about world issues in an age-appropriate manner, and in ways that help to facilitate conversation. Furthermore, *BtN* has provided tools for parents and guardians during the COVID-19 pandemic, modelling appropriate amounts and types of information for children to receive about the crisis.

While *BtN* is broadcast on the ABC, this example also highlights the role of commercial networks in the educational Australian children’s television ecology. When *BtN* was cancelled in 2003, commercial broadcaster Channel 10 launched a similar programme called *the total news* (*ttn*) in 2004. Initially produced in response to *BtN*’*s* controversial cancellation, *ttn* continued to air after the 2005 re-launch of *BtN* for another 5 years. While *BtN* is aimed at primary school students, *ttn* was directed towards a high-school demographic. Reflecting on *ttn*’*s* cancellation, producer and presenter Scott Beveridge (2009) asserted, ‘having more than one kids news service makes sense, not just to give the early high schoolers an alternative, but to give teachers a choice’ (p. 77). The aforementioned programme the ABC recently cancelled due to budget cuts, *Defintely Not news (DNN)*, highlights the evolution of the TV news genre for young people in Australia. Launched during the COVID-19 pandemic, *DNN* playfully parodied news and current affairs television in ways that aimed to help alleviate the social isolation anxiety of young viewers (TV Black Box, 2020). In an era of increasingly globalised current affairs content and ‘fake news’ across a multitude of digital platforms, there is arguably an even greater need for a diversity of reliable local news services that cater to a range of youth demographics.

## *Bluey*, everyday knowledge and learning through play

Beyond curricula and news, Australian children’s content provides less formalised ‘everyday knowledge’ (Gardiner, 2006: 205) that is attentive to local specificities. This is a role of Australian television content that is particularly important for younger children who have not yet started school. Highlighting this issue, in 2012, an episode of the British animated series *Peppa Pig* in which the pink protagonist befriends a spider was removed from circulation by the ABC. Given the many dangerous arachnids found in Australia, viewers complained that the episode was ‘inappropriate for an Australian audience because it said that spiders were not to be feared’ (Zhou, 2017). By contrast, the local animated series *Bluey* – initially conceived as an ‘Australian version of *Peppa Pig*’ (Collins, 2019) – has received acclaim for conveying locally rooted everyday knowledge in engaging ways for both young children and their parents.

*Bluey* follows the daily lives of a family of anthropomorphised Australian Cattle Dogs: a 6-year-old blue heeler Bluey, her younger sister Bingo and their parents Bandit and Chilli. The show is set in semi-tropical North Queensland and is stocked with signifiers of everyday Australian life, including native wildlife, backyard BBQs and ‘verandah Santas’. Commenting on the show’s importance in a pre-school landscape increasingly dominated by overseas content, curriculum officer at the ACTF, Janine Kelly (2018), writes, ‘for many children, *Bluey* is the first distinctly Australian animation they will see. It will contribute to their perceptions of Australian life and their developing national identity’. Examples such as *Bluey* illustrate Potter’s (2015) argument that ‘much of the worth’ of Australian children’s television ‘lies in its ability to situate children within their own culture’ (p. ix).

*Bluey*’*s* second season launched on 17 March 2020, a few days before the Federal Government introduced strict social distancing measures in response to the COVID-19 pandemic. The show’s return at a time when families were housebound was described as a ‘welcome relief’ by Screen Queensland’s CEO Kylie Munnich (Goulis, 2020). Like other locally produced pre-school shows, such as *The Wiggles* and *Play School, Bluey* endeavoured to educate children about good hygiene during the pandemic with a specially produced downloadable poster.^2^

**Figure 1.**
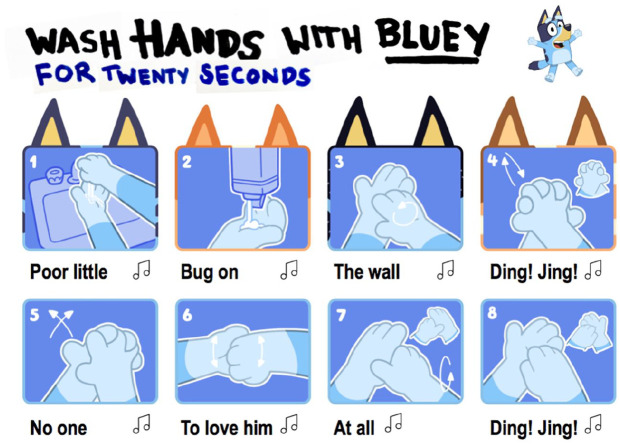
A section from the *Bluey* Sing-A-Long Hand Washing Poster released in response to COVID-19.

Figure 1 presents a section from the *Bluey* Sing-A-Long Hand Washing Poster.

The pedagogical function of *Bluey* extends beyond such ‘everyday knowledge’. While the show emphasises its Australian status, *Bluey* challenges some problematic tenets of Australian national identity. In one backyard set episode, *BBQ*, Dad Bandit is cooking at the barbeque, which primarily involves drinking and chatting, while Mum Chilli is seen in the background managing the children and setting up for their guests. When Bandit’s delivery of sausages to the dinner table is met with cheers from the family and guests, the youngest child, Bingo, reminds everyone to notice the salad (and by implication Chilli’s work). Bingo’s comment prompts enthusiastic recognition of Chilli’s contribution and a knowing look between mother and daughter. Moments such as this acknowledge the gender dynamics and disparities that go into making a ‘classically’ Australian scene. As Isabella Steyer (2004) cautions of gender representation in children’s media, sexist portrayals ‘may affect children’s development in a number of ways and lead to a reproduction of gender stereotypes’ (p. 171).

*Bluey* has also been praised for ‘capturing the wonder and joy of parenting, and delivering precious nuggets of parenting wisdom along the way’ (Whittingham et al., 2019). Creator Joe Brumm points out that the show is designed to be ‘co-viewed’ with both parents and children. As he explains, the show aims to model ‘learning through play’ in ways instructive for adults as well as their children, to ‘show parents that the kids aren’t just mucking around’ when they are playing (cited in Collins, 2019). Most episodes feature one or both parents as eager participants in children’s games such as a ‘Mount Mumanddad’, ‘Daddy Robot’, and ‘Horse Wedding’ with Bandit and Uncle Stripe as the bride and groom. As Whittingham et al. (2019) note, these games impart advice to parents who may be watching that is ‘remarkably consistent with the scientific literature on parenting and parental wellbeing’. Similarly, journalist and parent, Andrew Street (2020) describes how *Bluey* informed his reaction to the pandemic, ‘I did what any right-thinking Australian father would do. I asked myself, “What would Bandit do?”’. The COVID-19 crisis put extensive demands on parents, with many relying on video content to entertain their children. Australian parents credited *Bluey* with providing tools to navigate the crisis, with Street (2020) explaining that the show ‘reminds us that it’s downright important for us to throw ourselves into play, because first, that is how children learn and second, our kids look to their parents as models for their own behaviour’.

## Conclusion

The case studies considered in this essay are a snapshot of the ways that the Australian children’s television sector has delivered culturally specific forms of education to Australian children. During the pandemic, this well-established but often overlooked activity enabled the sector to pivot to support children’s learning and the pedagogical aims of caregivers, parents and teachers in ways that responded to the COVID-19 crisis. As debates continue about how best to regulate Australian children’s TV in an increasingly digitalised and globalised media environment, it is important to recognise these contributions. The socio-cultural value of Australian children’s television has been accentuated during the pandemic, yet at the same time the infrastructure that buttresses the sector is now under threat.

## Notes

1. The programme’s hiatus was partly related to Federal Government budget cuts to the ABC in 2003. Pavolic (2018) references protest staged by teachers and students when the show was cancelled, quoting a primary school student who declared ‘How could they do this to me? I have rights!’

2. In response to the COVID-19 pandemic, the Australian children’s music group *The Wiggles* posted a ‘Social Distancing’ song online on 29 March 2020, in which children were invited to become a ‘hand washing hero’ through good hygiene. Similarly, a COVID-19 special of Australian educational television show *Play School* was produced that sought to ‘explain some of the big questions around coronavirus’.
